# Validation and cross-cultural adaptation of the Bedside Language test for Brazilian patients

**DOI:** 10.1590/1980-5764-DN-2025-0445

**Published:** 2026-07-20

**Authors:** Milena Gama Setúbal Freitas, Ingrid César Fernandes, Gustavo Cavalcante Cruz de Almeida, Artur Victor Menezes Sousa

**Affiliations:** 1 Hospital Geral de Fortaleza, Departamento de Neurologia, Fortaleza CE, Brazil.

**Keywords:** Aphasia, Stroke, Language Tests, Validation Study, Reproducibility of Results, Afasia, Acidente Vascular Cerebral, Testes de Linguagem, Estudos de Validação, Reprodutibilidade dos Testes

## Abstract

Aphasia is a neurological condition characterized by partial or total loss of verbal communication, most commonly caused by stroke. In hospital settings, brief and reliable tools are essential to identify and quantify aphasia early, enabling prompt diagnosis and appropriate rehabilitation planning. However, Brazil lacks language assessment instruments adapted for bedside use.

**Objective:**

To perform the cross-cultural adaptation and validation of the Bedside Language test for Brazilian Portuguese.

**Methods:**

The BL underwent translation, back-translation, expert review, and cultural adaptation. The final version was administered to 70 participants. Two evaluators applied the instrument on the same day to assess inter-rater reliability, and the same examiner reapplied it within two days to assess test-retest reliability. The Bedside Evaluation Screening Test (BEST-2) language test was also used for concurrent validity.

**Results:**

The Brazilian version of the BL demonstrated high internal consistency (Cronbach's alpha: spontaneous reading — LE=0.80; comprehension — CO=0.86; repetition — RE=0.95; writing — ES=0.73; overall=0.87), as well as satisfactory intra-rater (p=0.22) and inter-rater (p=0.77) reliability. It showed a strong correlation with the BEST-2 across all linguistic domains except writing.

**Conclusion:**

The Brazilian Portuguese version of the BL is a brief, valid, and reliable bedside screening tool for identifying aphasia in patients with ischemic stroke, supporting high-quality care in specialized stroke centers.

## RESUMO

A afasia é uma condição neurológica caracterizada pela perda parcial ou total da capacidade de comunicação verbal, cuja causa mais comum é o acidente vascular cerebral (AVC). No contexto hospitalar, são essenciais instrumentos breves e confiáveis para identificar e quantificar a afasia precocemente, favorecendo o diagnóstico rápido e o planejamento adequado da reabilitação. No Brasil, há escassez de instrumentos de avaliação linguística adaptados para uso à beira-leito.

**Objetivo:**

Realizar a adaptação transcultural e validação do Bedside Language test para o português do Brasil.

**Métodos:**

O BL passou por etapas de tradução, retrotradução, análise por especialistas e adaptação cultural. A versão final foi aplicada a 70 participantes. Dois avaliadores aplicaram o instrumento no mesmo dia (para avaliar a confiabilidade interavaliadores), e o mesmo pesquisador o reaplicou após dois dias (para avaliar a confiabilidade teste-reteste). O teste Bedside Evaluation Screening Test (BEST-2) foi utilizado para análise de validade concorrente.

**Resultados:**

A versão brasileira do BL apresentou alta consistência interna (alfa de Cronbach: leitura espontânea — LE=0,80; compreensão — CO=0,86; repetição — RE=0,95; escrita — ES=0,73; total=0,87), bem como confiabilidade intra-avaliador (p=0,22) e interavaliadores (p=0,77) satisfatória. Observou-se forte correlação com o BEST-2 em todos os domínios linguísticos, exceto escrita.

**Conclusão:**

A versão em português brasileiro do BL mostrou-se um instrumento breve, válido e confiável para o rastreio de afasia em pacientes com AVC isquêmico, apoiando o cuidado de qualidade em centros especializados em AVC.

## INTRODUCTION

Stroke remains one of the leading causes of mortality and long-term disability worldwide and represents a major public health challenge, particularly in low- and middle-income countries^1^. In Brazil, despite a reduction in mortality rates over recent decades, stroke continues to be a primary cause of functional impairment, generating significant social, economic, and healthcare burdens^2^. Among the cognitive sequelae of stroke, aphasia is one of the most frequent and clinically relevant conditions, affecting the ability to comprehend and produce spoken and written language to varying degrees^3^. Aphasia differs from motor speech disorders such as dysarthria or apraxia of speech, as it primarily involves disruptions in language processing rather than speech execution^4,5^. In acute and subacute hospital settings, aphasia may be one of the earliest and most functionally disabling manifestations of stroke, directly impacting communication, clinical decision-making, and ­rehabilitation planning^4^.

Early identification of language impairments in hospitalized stroke patients is therefore essential. In this context, bedside screening instruments play a critical role by enabling rapid, structured, and reproducible assessment of language functioning during the acute phase, when comprehensive neuropsychological or speech-language evaluations may be impractical or contraindicated^4^. Screening tools are not intended to replace formal diagnostic batteries but to support early detection, referral, and monitoring of language deficits in complex clinical environments.

From a clinical perspective, aphasia encompasses a heterogeneous group of syndromes resulting from lesions affecting the dominant hemisphere language network, most commonly following ischemic stroke. These syndromes may vary in severity and presentation, frequently involving impairments across multiple linguistic domains and often co-occurring with other neurological deficits (Table 1)^6-16^. In real-world hospital practice, however, patients do not always conform neatly to classical aphasia subtypes, reinforcing the need for brief instruments that focus on functional language abilities rather than strict syndromic classification^3^.

**Table 1:** Main types of aphasia: lesion location, clinical features, and impaired/preserved functions.

Types of aphasia	Lesion location	Type	Clinical features	Preserved functions	Impaired functions
Broca's (motor, expressive)	Broca's area (left inferior frontal region) — center responsible for speech motor execution and sentence formation^6^	Non-fluent^7,8^	Interrupted speech, effortful speech, verbal stereotypies (words or phonemes)^6^	Oral comprehension^6^	Spontaneous speech, reading, writing, naming^8^
Wernicke's (sensory)	Wernicke's area(Brodmann area 22) — center responsible for word comprehension and planning^9^	Fluent^6^	Fluent but meaningless speech, paraphasias, anosognosia. Generally, no associated motor deficits and patients appear physically healthy^10^	Speech fluency^11^	Comprehension, repetition, reading, writing^11^
Conduction	Arcuate fasciculus — neural pathway connecting Wernicke's area to Broca's area^12^	Fluent^11^	Difficulty in repetition, recognizing errors, and attempting to correct them^12^	Comprehension, relative fluency^12^	Repetition, writing (with paraphasic errors)^12^
Transcortical motor	Region anterior to Broca's area, or in adjacent subcortical areas at the border between the middle cerebral artery and the anterior cerebral artery^12^	Non-fluent^13^	Minimal spontaneous speech, speech composed of automatic words (expressions frequently used throughout the patient's life)^6^	Repetition^13^	Speech fluency, spontaneous language^13^
Transcortical sensory	Areas surrounding Wernicke's area^12^	Fluent^12^	Fluent speech, impaired comprehension, repetition preserved^12^	Repetition^14^	Comprehension, lexical-semantic association^12^
Mixed transcortical	Areas surrounding Broca's, Wernicke's, and the arcuate fasciculus (isolation syndrome of the language area)^15^	Mixed^15^	Severe impairment of speech and comprehension, presence of echolalia^15^	Repetition^15^	Spontaneous speech, comprehension, naming^15^
Global	Extensive lesion in the perisylvian area (territory of the left middle cerebral artery)^13^	Non-fluent^13^	Severe deficits in all language functions, speech limited to sounds or few words^15^	–	Comprehension, speech, reading, writing, naming, repetition^15^
Anomic	Angular gyrus^13^	Fluent^13^	Word-finding difficulties, vague speech^16^	Comprehension, fluency, reading, writing^16^	Naming (lexical access)^16^

Source: Prepared by the author; 2025^6-16^.

Despite the high prevalence of stroke-related aphasia, Brazil lacks validated language screening instruments specifically adapted for bedside use in acute hospital settings. Many available tools are either not culturally adapted, not standardized, nor designed for outpatient or research contexts. This gap limits early identification of language impairments and may delay appropriate referral for rehabilitation within the public healthcare system^17^.

The Bedside Language^4^ test (BL), originally developed in Argentina, is a brief screening instrument designed to assess core language domains in hospitalized patients with suspected aphasia. Its short administration time and structured format make it particularly suitable for acute care environments. Given the linguistic and cultural differences between Spanish and Brazilian Portuguese, as well as the specific demands of the Brazilian healthcare context, cross-cultural adaptation and psychometric validation of the BL are necessary before its clinical application.

Thus, the present study aimed to perform the cross-cultural adaptation of the BL test for Brazilian Portuguese, and to examine its reliability and validity as a bedside screening instrument for aphasia in patients with ischemic stroke treated in a high-complexity hospital setting.

## METHODS

The cross-cultural validation of the test was conducted in a cross-sectional study with 70 participants who had brain lesions due to ischemic stroke. The experimental group included 31 individuals with aphasia resulting from left hemisphere lesions, while the control group comprised 17 individuals with dysarthria due to right hemisphere lesions and 22 individuals without speech or language impairments, with lesions in either hemisphere (Table 2). This design allowed for the assessment of the test's ability to discriminate between aphasia and other communication disorders.

**Table 2:** Demographic variables of the groups.

	Aphasia group (n=31)	Dysarthria group (n=17)	No speech/language impairment (n=22)
Men, n (%)	16 (22.8)	11 (15.7)	15 (21.4)
Women, n (%)	15 (21.4)	6 (8.57)	7 (10)
Age (years)	52.6 (15; 81)	58.6 (26; 76)	49.6 (19; 72)
Mean education (years)	8.38 (2;17)	10.41 (3;17)	10.22 (2;17)

Source: Prepared by the author; 2025.

The sample was recruited by convenience from two units of a specialized neurology hospital: Ischemic Stroke Unit (n=56) and Neurology Ward (n=14). Inclusion criteria were:

- vascular lesion confirmed by neuroimaging;
- no prior psychiatric history before the stroke, confirmed through family interview and medical record review; and
- education compatible with reading and writing tasks.

Previous diagnoses of aphasia or dysarthria were established by the clinical team (physicians and speech language pathologist).

In the aphasia group, 26 participants presented non-fluent aphasias (global: 18; Broca: 4; transcortical motor: 2; mixed transcortical: 2), and five participants presented fluent aphasias (anomic: 2; Wernicke: 2; conduction: 1). The predominance of global aphasia is consistent with the literature on the topic^18^. All patients underwent computed tomography for lesion confirmation.

### Ethical approval

The study was approved by the Ethics Committee of Hospital Geral de Fortaleza (Certificate of Presentation for Ethical Appreciation — CAAE n^o^ 79751924.4.0000.5040), and the original author of the BL test authorized the ­adaptation. All participants, or their legal guardians, signed an informed consent form. The validation process involved four steps: translation, cross-cultural adaptation, reliability analysis, and validity assessment.

### Translation

Translation followed four steps:

1. Two bilingual translators, native in Brazilian Portuguese, independently translated the instrument.
2. The versions were compared and merged into a single version.
3. This version was back-translated by two native Spanish speakers.
4. The back-translations were compared with the ­original, and after consensus among the researchers, a provisional Brazilian Portuguese version was defined.

### Cross-cultural adaptation

Cultural aspects were considered to ensure interpretive equivalence. The Brazilian version was applied in a pilot phase with ten ischemic stroke patients at the hospital where the research was conducted. Adjustments were made based on participants’ responses to ensure clarity and comprehension. The final version is presented in Supplementary Material.

### Reliability

Reliability was assessed through temporal consistency (test-retest), inter-rater equivalence, and internal consistency (homogeneity). The test was administered twice by different psychologists, with an interval of up to two days.

### Validity

Validity was examined by comparing the results of the Brazilian version of the BL with those of the Bedside Evaluation Screening Test — 2nd Edition (BEST-2), ­validated in Brazil. The BL assesses five language domains: spontaneous language, oral comprehension, repetition, reading, and writing.

### Group distribution

**Figure 1:**
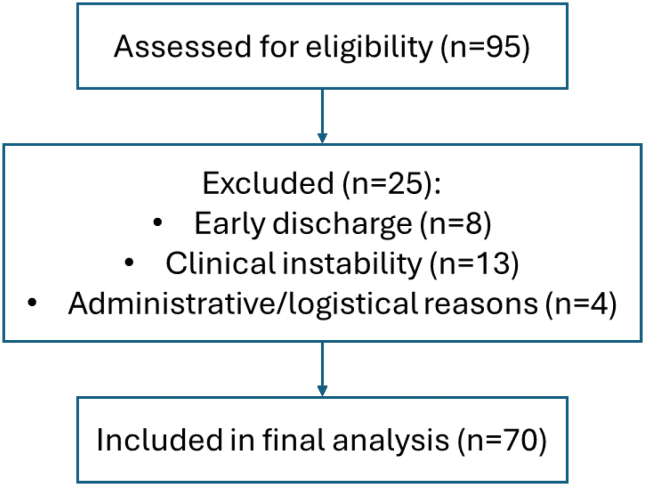
Flow diagram of participant inclusion and exclusion.

Source: Prepared by the author; 2025.

After the pilot study, the instrument was applied to 95 participants distributed into three groups: controls (post-stroke without language alterations), stroke with aphasia, and stroke with dysarthria. Of these, 25 were excluded for clinical, administrative reasons, or early discharge, resulting in 70 participants included across the three study phases (Figure 1).

### Clinical and neuropsychological assessment

Language assessment was conducted using two instruments: the Brazilian version of BL^4^ and the BEST-2^19^. The BL, developed by Argentine researchers, is a brief test (∼10 minutes) assessing five language domains: spontaneous language, comprehension, repetition, reading, and writing.

For spontaneous language, participants provide personal information and describe an image (maximum 5 points). Comprehension is assessed with yes/no questions and commands of increasing complexity (maximum 6 points). Repetition includes two words and three phrases (5 points). Writing includes full name, dictation of a word or number, and written production from the described image (5 points). Motor limitations were accommodated using movable letters. Reading tasks include word/object matching, reading simple commands, and filling in sentences (5 points). The maximum total score is 26.

The Brazilian version retained the original structure, with slight adaptation of the item "Estamos en Escobar?" to "Estamos no Brasil?" to ensure comprehension by Brazilian participants.

The BEST-2, validated in Brazil by Marchi^19^, served as a comparative instrument. It evaluates conversation, naming, repetition, reading, and command comprehension and is recognized for its effectiveness in identifying aphasic syndromes.

To investigate internal structure of the BL, a parallel analysis was conducted, suggesting a single-factor retention, differing from the theoretical hypothesis^20^. Exploratory factor analyses (EFA) were performed by item group using principal axis factoring (PAF) without rotation in the Statistical Package for the Social Sciences — SPSS (v.25). Reliability was estimated using Cronbach's alpha, Guttman's lambda, McDonald's omega, and the Composite Reliability Index.

External validity was assessed through Student's t-tests, comparing groups with and without language impairments, with effect size calculation (Cohen's d). Correlations between BL domains and external measures were also analyzed to verify convergence and strengthen the instrument's validity.

## RESULTS

Table 3 presents the data for the evaluated groups (­aphasia — AG; dysarthria — DG; no impairment — NIG) across BL subscales: spontaneous language, comprehension, repetition, writing, and reading.

**Table 3:** Group Comparison Results — Bedside Language Test (1^st^ Administration).

Domain	Group	Mean	SD	Median	Min	Max	n
Spontaneous language	AG	1.20	1.56	0.00	0.0	4.5	31
	DG	5.00	0.00	5.00	5.0	5.0	17
	NIG	4.91	0.43	5.00	3.0	5.0	22
Comprehension	AG	2.98	2.15	3.00	0.0	4.5	31
	DG	5.82	0.53	6.00	4.0	6.0	17
	NIG	5.91	0.43	6.00	4.0	6.0	22
Repetition	AG	1.9	2.04	2.00	0.0	5.0	31
	DG	5.0	0.00	5.00	5.0	5.0	17
	NIG	5.0	0.00	5.00	5.0	5.0	22
Writing	AG	0.87	1.20	0.00	0.0	4.0	31
	DG	4.24	1.53	5.00	0.0	5.0	17
	NIG	4.95	0.21	5.00	4.0	5.0	22
Reading	AG	1.82	1.97	1.75	0.0	5.0	31
	DG	4.88	0.49	5.00	3.0	5.0	17
	NIG	5.00	0.00	5.00	5.0	5.0	22

Abbreviations: SD, standard deviation; Min, minimum; Max, maximum; AG, aphasia; DG, dysarthria; NIG, no impairment.

Source: Prepared by the author; 2025.

### Factor analysis

The BL was theoretically conceived as a multidimensional screening instrument, comprising distinct but interrelated language domains — spontaneous speech, comprehension, repetition, reading, and writing —, each representing specific functional components of language assessment in clinical settings. Within this framework, each domain was assumed to present local unidimensionality, while collectively reflecting a broader construct of general language functioning.

In line with theory-driven approaches to scale development and construct validation^21,22^, the internal structure of the BL was examined at the domain level, rather than through a single global EFA including all items simultaneously. This analytic strategy was adopted to preserve the theoretical and clinical interpretability of the predefined language domains, which comprise heterogeneous item content reflecting distinct linguistic functions.

Accordingly, EFAs conducted by theoretical item grouping revealed a consistent unifactorial structure within each BL dimension, supporting the assumption of local unidimensionality. In the spontaneous language domain, factor loadings ranged from 0.87 to 0.97 (explained variance=87.63%). In the comprehension domain, loadings ranged from 0.49 to 0.95 (explained variance=66.23%), with one item (BL04) presenting a comparatively lower loading. For the writing domain, factor loadings ranged from 0.85 to 0.93 (explained variance=78.65%). Finally, the reading domain showed factor loadings between 0.83 and 0.92 (explained variance=79.08%), indicating good internal cohesion across domains.

### Statistical assumptions and data inspection

Prior to inferential analyses, the distributional properties of the BL domain scores were inspected through visual examination of histograms and Q-Q plots, as well as descriptive indices of central tendency and dispersion. Homogeneity of variances between groups was evaluated using Levene's test when parametric group comparisons were performed. Given the clinical nature of the sample and the presence of ceiling effects in some domains, departures from normality were anticipated. When assumptions of normality or homoscedasticity were not fully met, the robustness of the applied statistical tests was considered in the interpretation of results, and effect sizes were emphasized alongside p-values.

### Internal reliability

Internal consistency was assessed using multiple coefficients: Cronbach's *α* (>0.7), Guttman's *λ* (0.88–0.93), McDonald's Ω (>0.7), and ICC (>0.7), showing good results across all dimensions: spontaneous language (*α*=0.80), comprehension (*α*=0.86), repetition (*α*=0.95), writing (*α*=0.73), and reading (*α*=0.87). These values indicate high internal consistency without excessive redundancy (Table 4).

**Table 4:** Internal reliability coefficients of bedside language dimensions.

Dimension	α	λ	Ω	ICC
Spontaneous language	0.80	0.93	0.96	0.95
Comprehension	0.86	0.90	0.93	0.90
Repetition	0.95	0.96	0.95	0.95
Writing	0.73	0.88	0.88	0.91
Reading	0.87	0.88	0.90	0.91

Abbreviation: ICC, intraclass correlation coefficient.

Source: Prepared by the author; 2025.

### Test-retest and inter-rater reproducibility

The BL scale demonstrated temporal stability and inter-rater reliability, with paired Student's t-tests showing no significant differences between T1, T2, and T3 (p>0.05). Intraclass correlation coefficient (ICC) values confirmed good reproducibility both intra- and inter-rater (Table 5).

**Table 5:** Bedside Language scale comparison over time.

Comparison	t	df	p-value
T1 vs T2	0.29	51	0.771
T1 vs T3	-1.23	55	0.223
T2 vs T3	-1.02	51	0.312

Abbreviation: df, degree of freedom.

Source: Prepared by the author; 2025.

Participants with aphasia scored significantly lower than the other groups (Cohen's d=1.83–4.49; p<0.001). The large effect size in the writing task (d=4.49) may have been inflated due to lack of variability in the control group, warranting cautious interpretation. No significant differences were observed between the dysarthria and control groups, consistent with expected clinical findings (Table 6).

**Table 6:** Mean differences between control and aphasia groups across Bedside Language dimensions and effect size.

Dimension	Control (n=22)	Aphasia (n=31)	Cohen's d
Spontaneous language	1.63 (0.14)	0.38 (0.51)	3.11*
Comprehension	1.18 (0.08)	0.58 (0.42)	1.83*
Repetition	1.00 (0.00)	0.36 (0.40)	2.08*
Writing	1.65 (0.21)	0.29 (0.39)	4.49*
Reading	1.66 (0.00)	0.58 (0.65)	2.16*
Total	1.42 (0.03)	0.44 (0.39)	3.26*

Note:

* p<0.001

Source: Prepared by the author; 2025.

### Criterion validity

Criterion validity was examined via Spearman correlations between the BL and BEST-2, with results ranging from 0.38 to 0.62 (p<0.001), indicating significant positive correlations across assessed domains.

## DISCUSSION

Early diagnosis of aphasia is crucial for neuropsycholinguistic rehabilitation, requiring instruments that are both rapid and sensitive to changes in the first days poststroke^4^. Screening tools are, therefore, valuable resources for identifying cognitive and language deficits during the acute phase, facilitating timely and appropriate referrals. Considering the high incidence of aphasia in Brazil, adapting an instrument such as the BL to the Brazilian sociocultural context becomes essential^21^.

This study emerged from the author's clinical practice in a hospital setting, where the need for standardized instruments to assess bedside communication was identified. Based on this need and considering the characteristics of the BL as a brief instrument, its cross-cultural adaptation and psychometric evaluation were conducted in patients with acute ischemic stroke.

The results indicate that the Brazilian version of the BL is psychometrically robust, sensitive, and specific for detecting aphasias, reliably distinguishing them from conditions such as dysarthria. The unidimensional factorial structure found in each domain confirms that the items in each subscale accurately assess the five main language components: spontaneous language, comprehension, repetition, reading, and writing — in line with the classical Wernicke-Lichtheim model.

Internal consistency was high (Cronbach's *α* ranging from 0.73 to 0.95), reinforcing its reliability even in challenging hospital contexts. Rapid assessments are crucial for early identification of aphasias and to guide referrals and prognoses. Comparisons between groups demonstrated the strong discriminative capacity of the BL, with significantly lower scores in the aphasia group and large effect sizes (Cohen's d=1.83–4.49). However, the extreme value observed in the writing task (d=4.49) should be interpreted with caution, as it may have been influenced by the lack of variability in the control group, which distorts the pooled standard deviation.

On the other hand, the absence of significant differences between the dysarthria and control groups reinforces the specificity of the instrument for language rather than motor impairments, which is essential for differential diagnosis. Temporal stability and inter-rater equivalence were confirmed, with stable scores across different time points and evaluators, supporting its use by various professionals within the hospital multidisciplinary team.

Convergent validity was also demonstrated, with moderate to strong correlations with the BEST-2. The observed moderate to strong correlations between the BL and the BEST-2 indicate meaningful convergence without redundancy, which is appropriate for a brief bedside screening tool. Differences in test structure, scope, and administration time likely account for the partial overlap observed, supporting the BL's complementary role in rapid clinical screening rather than equivalence to comprehensive assessments.

By preserving the original structure and making only minor cultural adaptations (e.g., "Estamos no Brasil?"), the Brazilian version demonstrated good linguistic and cultural sensitivity. Nonetheless, the small sample size is a limitation, attributable to operational difficulties in the hospital context, such as clinical instability, high turnover of admissions, and patient transfers — common factors in research conducted in high complexity settings.

The present findings should be interpreted within the clinical context in which the instrument was validated. The sample was predominantly composed of hospitalized patients in the acute or subacute phase of ischemic stroke, with a higher proportion of individuals presenting moderate to severe aphasia. Consequently, the psychometric properties and discriminative performance observed in this study are most directly applicable to moderate to severe post-stroke aphasia in hospital settings, where rapid bedside screening is clinically indicated. Caution is warranted when extrapolating these results to individuals with milder language impairments or to outpatient and chronic care populations, as the sensitivity and specificity of the instrument in these contexts were not directly examined in the present study.

The Brazilian BL offers important practical advantages: it is brief (∼10 minutes), bedside-applicable, and easy to use by different professionals, including in high-demand units. However, as a screening instrument, its assessment is superficial and does not replace formal test batteries. Cognitive or sensory deficits (e.g., apraxia, hemianopia) may interfere with results. Naming is not directly assessed, potentially limiting sensitivity, and the use of visual stimuli may challenge patients with perceptual impairments. Therefore, it is recommended to complement the BL with more specific instruments when necessary.

In summary, the findings suggest that the Brazilian BL is valid, reliable, and applicable in multidisciplinary clinical practice within the Unified Health System (Sistema Único de Saúde — SUS), serving as a promising tool for aphasia screening with the potential to accelerate diagnosis and optimize referral for rehabilitation.

The Brazilian Portuguese version of the BL test is made available for open clinical and research use, with the aim of facilitating early identification of aphasia in hospital settings. Authorization for cross-cultural adaptation was obtained from the original author, and no additional permission is required for the clinical application of the instrument beyond appropriate citation.

In conclusion, this study adapted and validated the BL test for Brazilian Portuguese, demonstrating that the Brazilian version is psychometrically robust, with a consistent factorial structure, high reliability, and good temporal stability. The BL proved effective in screening for aphasia in patients with ischemic stroke, distinguishing language impairments from other conditions such as dysarthria.

The application was feasible in a hospital setting and useful for various professionals, supporting clinical decision-making and referral for rehabilitation. Despite the sample size limitation and the brief nature of the instrument, the BL demonstrated validity and functionality in clinical practice. Future studies are recommended to expand its applicability within the SUS.

## Data Availability

The datasets generated and/or analyzed during the current study are not publicly available due to ethical and privacy restrictions, but are available from the corresponding author upon reasonable request.
