# Supplementary material for: Validation and cross-cultural adaptation of the Bedside Language test for Brazilian patients
Source: Dement Neuropsychol. 2026 Jul 20;20:e20250445. doi: 10.1590/1980-5764-DN-2025-0445 (PMC13387805; doi:10.1590/1980-5764-DN-2025-0445)
Supplement: Supplementary Material [file 1980-5764-dn-20-e20250445-Suppl01.pdf]

BL - Teste de rastreio de afasias, versão brasileira

(Versão original: Bedside de Language, Sabe *et al.*, 2008)

Pontuação

**Liguagem Espontânea**	
**Compreensão**	
**Repetição**	
**Escrita**	
**Leitura**	

__________ **/26**

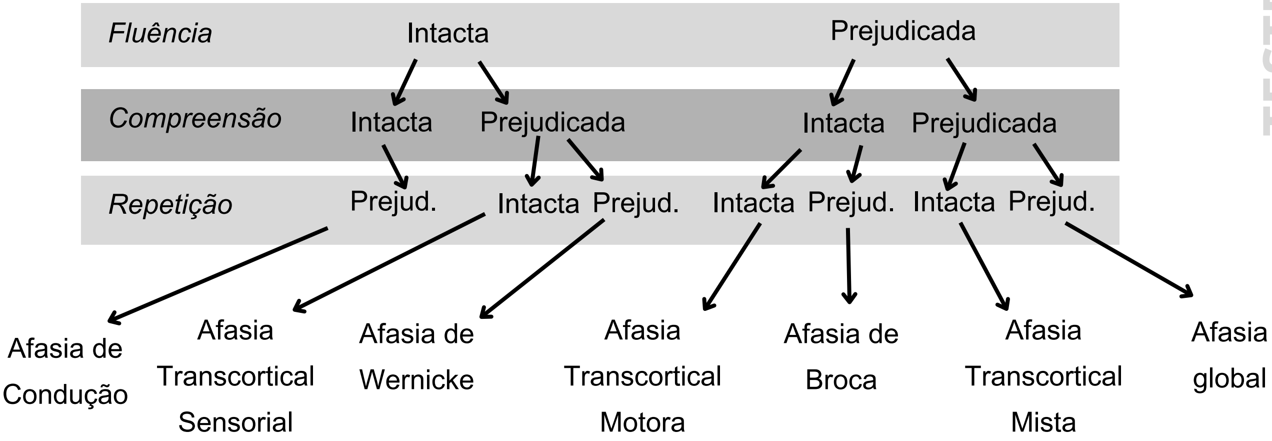

Conclusões

____.

Freitas, M.G.S.; Sousa, A.V.M.; Fernandes, I.C.; Almeida, G.C.C., 2025.

BL - Teste de rastreio de afasias, versão brasileira

(Versão original: Bedside de Language, Sabe *et al*., 2008)

A avaliação do BL é constituída por papel e lápis (ou por um quadro magnético com letras, números e desenhos, opcionalmente), alguns objetos reais ou imagens dos objetos. Os subtestes do BL são classificados em ordem de complexidade. O examinador deve suspender a avaliação nos casos em que o paciente obtiver 0 nos primeiros itens.

Nome: ____

Idade: _________ Escolaridade: ____________Lateralidade: ____________

Profissão: ________________________ Comorbidades: __________________________

Data: __/__/__ Examinador(a): __________________ Profissão: ___________________

Linguagem Espontânea

Nesta seção, o paciente é questionado sobre seu nome e endereço e, em seguida, é mostrada uma imagem de “um senhor lendo um jornal’, com a instrução “diga tudo o que você vê nesta imagem’.

**Pontuação**	
	Nome<br>0 pontos: ausência de fala ou compreensão auditiva.<br>0,5 pontos: resposta incompleta ou parafasias fonêmicas.<br>1 ponto: responde corretamente nome e sobrenome.
	Endereço<br>0 pontos: ausência de fala ou compreensão auditiva (estereotipias, etc).<br>0,5 pontos: reposta incompleta ou parafasias fonêmicas.<br>1 ponto: responde com seu endereço completo.
	Descrição da imagem 0 pontos: ausência de fala ou compreensão auditiva.<br>1 ponto: apenas uma unidade de conteúdo.<br>2 pontos: duas unidades de conteúdo.<br>3 pontos: estrutura sintática completa.
_________ **/5**	

TESTE BREVE PARA RASTREIO DE AFASIAS

Compreensão

Esse subteste é dividido em duas partes: a primeira refere-se a questões nas quais o paciente deve responder sim ou não. Elas estão relacionadas ao contexto atual. A segunda são ordens simples de complexidade crescente até três comandos. Elas nos permitem avaliar a capacidade do paciente em processar a informação auditiva. É importante levar em conta que as afasias muitas vezes são acompanhadas de apraxia, portanto, para poder aplicar este item, é necessário que a capacidade de realizar movimentos voluntários esteja preservada. Cada pergunta ou comando deve ser repetido completamente apenas uma vez, e só será repetido completamente se o paciente assim o requerer (em caso de déficit de atenção grave).

**Pontuação**	
	Estamos no Brasil?<br>O pontos: resposta errada (caso o paciente esteja desorientado no espaço, outra pergunta semelhante pode ser feita).<br>1 ponto: resposta correta.
	Você está usando uma jaqueta vermelha?<br>O pontos: resposta incorreta.<br>1 ponto: resposta correta.
	Toque no seu nariz<br>O pontos: execução incorreta.<br>1 ponto: execução correta.
	Aponte para a cadeira e depois para a porta<br>O pontos: execução incorreta.<br>0,5 pontos: execução incompleta.<br>1 ponto: execução completa.
	Olhe para a porta, olhe para mim e depois feche os olhos<br>0 pontos: execução incorreta.<br>0,5 pontos: execução incompleta.<br>1 ponto: erro na ordem.<br>2 pontos: execução correta.
__________ **/6**	

Repetição

Neste subteste se evalia a repetição de palavras e frases curtas. O único propósito de incluir essa variável nesta bateria é que ela adiciona uma característica diagnóstica distinta que ajuda a identificar afasia de condução e afasias transcorticais.

Pontuação	
	Pão<br>O pontos: ausência de resposta ou parafasias.<br>1 ponto: repeticão correta.
	Rádio<br>O pontos: ausência de resposta ou parafasias.<br>1 ponto: repetição correta.
	Algumas vezes<br>O pontos: ausência de resposta ou parafasias.<br>1 ponto: repetição correta.
	Fecha a porta!<br>O pontos: ausência de resposta ou parafasias.<br>1 ponto: repetição correta.
	Envie isso para seu amigo antes de quinta-feira<br>O pontos: ausência de resposta.<br>1 ponto: repetição correta.
__________ **/5**	

Escrita

O exame de escrita é análogo ao da linguagem espontânea, pois examinamos a mecânica dos movimentos da escrita, a evocação dos símbolos escritos para realizá- los através de diversos modos de estimulação, e a formulação de frases a partir de uma folha, e de um ditado. É feita uma análise de toda a produção escrita do paciente, que inclui o nome e o sobrenome, a escrita ditada de uma palavra e de um número de três dígitos, e a escrita de uma frase baseada na imagem mostrada anteriormente no item linguagem espontânea.

Pontuação	
	Nome e sobrenome<br>O pontos: ausência de letras legíveis.<br>0,5 pontos: resposta incompleta.<br>1 ponto: nome e sobrenome completo.
	Escrita por ditado (opção: letras móveis)<br>O pontos: ausência de letras ou números legíveis, ordem incorreta.<br>0,5 pontos: resposta incompleta.<br>1 ponto: execução correta em qualquer modalidade.
	Escrita de frase simples (imagem "um senhor lendo o jornal")<br>O pontos: ausência de palavras.<br>1 ponto: somente uma unidade de conteúdo.<br>2 pontos: duas unidades de conteúdo.<br>3 pontos: estrutura sintática completa.
_________ **/5**	

Leitura

Este subteste é dividido em três partes: na 1ª avalia-se a associação palavra- desenho: é apresentada uma folha com 3 palavras escritas, devendo o paciente colocar ao lado de cada palavra o respectivo desenho correspondente a ela; na 2ª parte o paciente deverá ler uma frase com uma ordem simples e executar o comando (é importante levar em consideração se existe apraxia associada); e na 3ª parte avalia-se a capacidade do paciente de completar frases com uma tarefa de escolha múltipla de quatro elementos.

**Pontuação**	
	Associação palavra-desenho ("xícara", "cadeira" e "sol"; quadro magnético com as palavras e as imagens para emparceirar)<br>O pontos: associação incorreta.<br>2 pontos: associação correta dos três estímulos.
	Comando simples escrito: 'levante a mão'<br>O pontos: ausência de execução.<br>1 ponto: execução correta.
	Frases para completar com opções<br>(A árvore tem ….. rodas, folhas, relva, fogo / O cão pode ….. correr, voar, ler, falar).<br>O pontos: execução incorreta.<br>1 ponto: escolha correta de uma das duas opções.<br>2 pontos: escolha correta de ambas orações.
__________ **/5**	

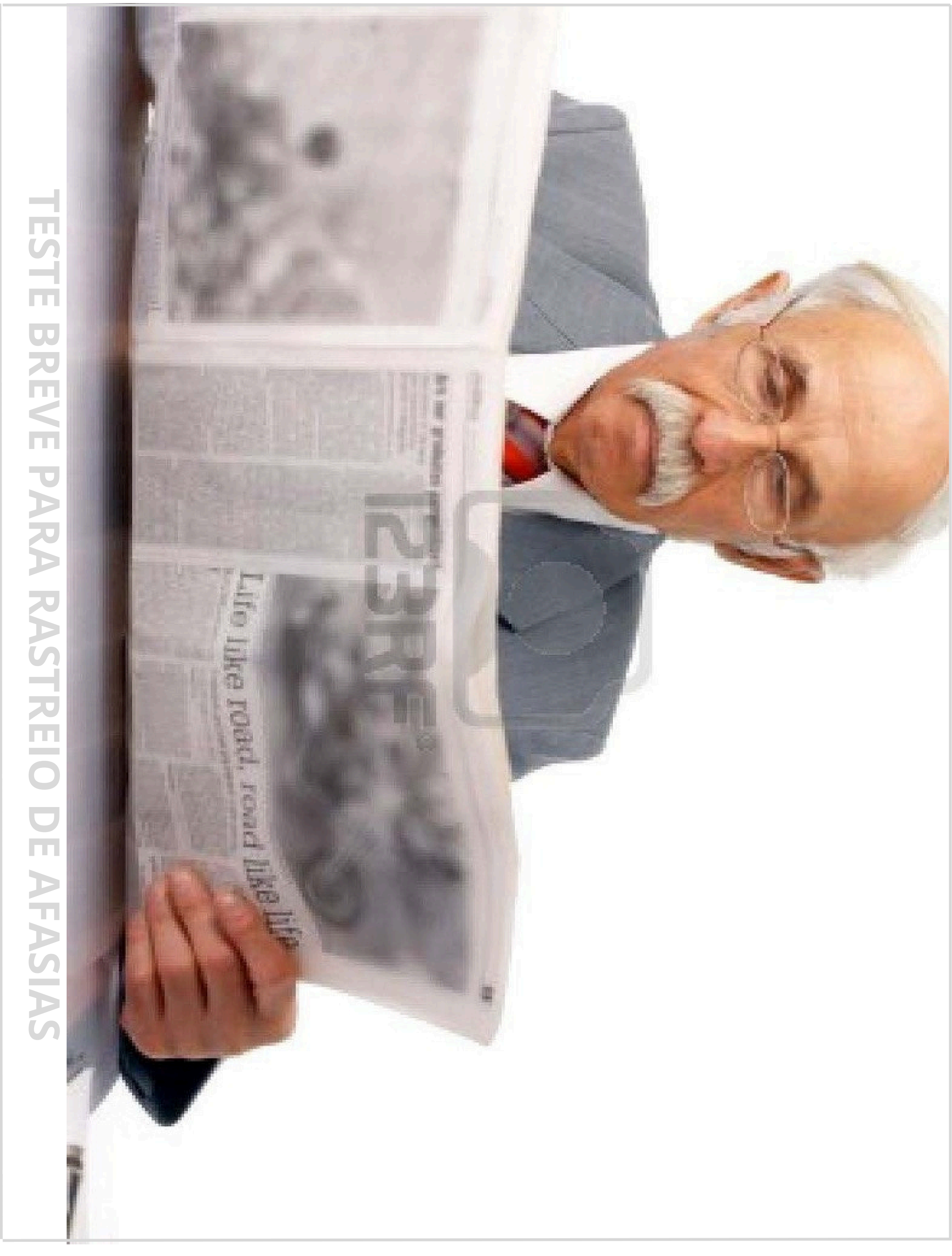
TESTE BREVE PARA RASTREIO DE AFASIAS

TESTE BREVE PARA RASTREIO DE AFASIAS

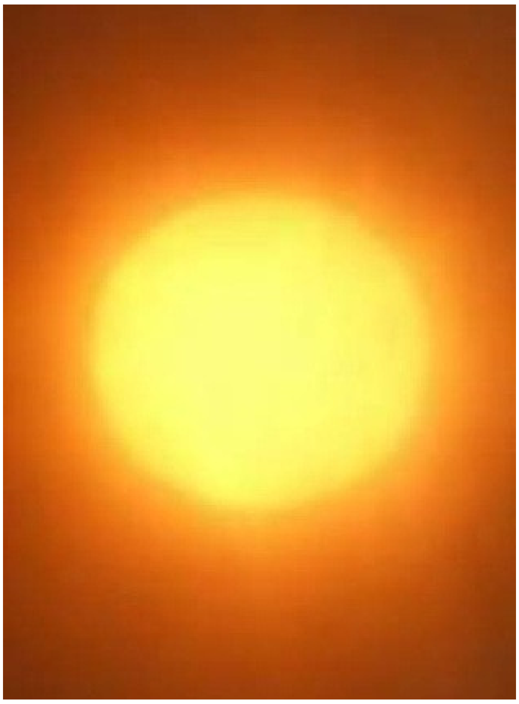

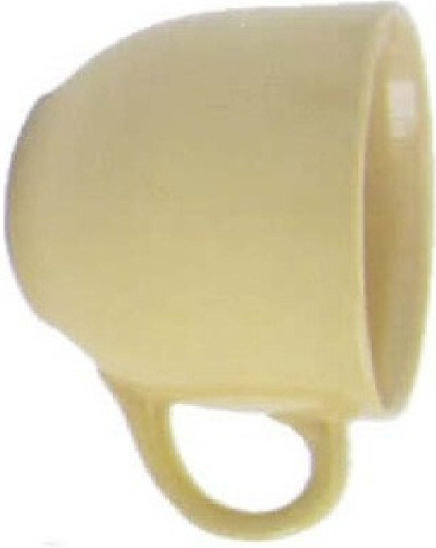

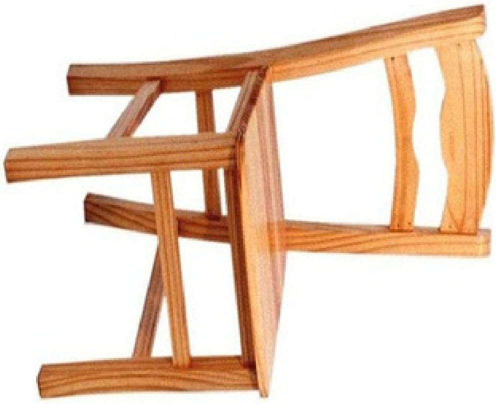

Xícara

Sol

Cadeira

Levante a mão

A árvore tem

rodas folhas relva fogo

O cachorro pode

correr voar ler falar
